# Genome-centric metagenomics reveals uncharacterised microbiomes in Angus cattle

**DOI:** 10.1038/s41597-025-04919-8

**Published:** 2025-04-01

**Authors:** Thibault P. R. A. Legrand, Pâmela A. Alexandre, Annaleise Wilson, Ryan J. Farr, Antonio Reverter, Stuart E. Denman

**Affiliations:** 1 CSIRO Agriculture & Food, St Lucia, Queensland Australia; 2 CSIRO Health & Biosecurity, Geelong, Victoria Australia

**Keywords:** Metagenomics, Microbiome, Applied microbiology

## Abstract

Understanding the intricate nexus between cattle health and microbiome dynamics holds profound implications for enhancing animal productivity and welfare. However, our understanding of the role of these microbial communities is limited in beef cattle, especially in understudied body sites such as the oral and nasal microbiome. Here, using a genome-centric metagenomics approach, we recovered substantial metagenome-assembled genomes (MAGs) from the faecal, oral and nasal microbiome of Australian Angus cattle from different herds and life stages. The MAGs recovered from faecal samples were dominated by Bacillota and Bacteroidota, while the MAGs from saliva and nasal mucus samples were mainly associated with Pseudomonadota, Actinomycetota and Bacteroidota. Functional annotation of the MAGs revealed enriched pathways involved in the production of some amino acids, nucleic acids and short chain fatty acids (SCFA). The metabolic capacities of the MAGs were correlated with their taxonomy, notably at the phylum level. Overall, this study provides a comprehensive catalogue of MAGs to further our understanding of their role in the health and fitness of beef cattle.

## Background & Summary

Cattle production is a cornerstone of the agricultural industry, encompassing the breeding, raising, and management of cattle for various purposes, including meat (beef), milk (dairy), leather, and other by-products. Beef cattle production is a vital segment dedicated to raising cattle for meat consumption and provides high-quality protein to a growing global population^1^. However, the beef cattle industry faces several hurdles that impact production, profitability, and sustainability. One of the main challenges is disease management, including several infectious diseases that can lead to substantial economic losses through reduced productivity and treatment costs^2^. Environmental concerns, such as methane emissions from cattle, also pose challenges in terms of regulatory compliance and sustainability goals. Microbiome studies offer a promising avenue to address these issues, by harnessing the power of microbial communities.

Advancements in next generation sequencing technologies have greatly deepened our knowledge on uncultivable microbes. Shotgun metagenome sequencing allows the discovery of unknown taxa by sequencing all genes in all organisms present in a given sample. First used at the beginning of the century, genome-resolved metagenomics allows the recovery of new microbial genomes by assembling and binning all sequenced reads into metagenome-assembled genomes (MAGs)^3^. This has led to the construction of microbial catalogues from various environments including animals, ocean, soil, plants and engineered environments^4^. Considering its importance in feed efficiency and methane emission, the rumen microbiome has been widely studied during the past few years which has led to the recovery of thousands of MAGs^5–10^. However, the microbial communities associated with other parts of the animal’s body are poorly understood, with only a few metagenomics studies investigating MAGs from other segments of ruminants’ gastrointestinal tract^11–14^. Despite its relevance for infectious diseases in cattle, studies exploring the nasal and oral microbiome have only been done using metabarcoding approaches which rely on already available microbial genomes^15–23^. Such approaches are limited to characterizing the structure of the microbial communities and as a result, there is a lack of information regarding their functional involvement and interactions with the host.

**Fig. 1:**
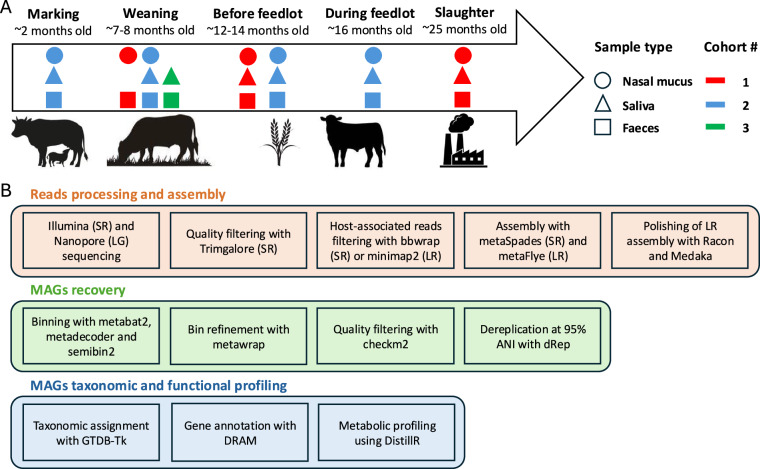
Sampling method and metagenomics workflow. (**A**) Diagram representing all samples collected in this study. Samples from various sample types were collected at five different life stage across three different herds. (**B**) Schematic representation of the bioinformatics pipeline used in this study to recover MAGs with further functional and taxonomic profiling.

In this study, we sequenced a total of 993 metagenome samples using short reads (SRs) Illumina sequencing which includes 377 from faeces, 325 from saliva and 291 from nasal mucus samples. In addition, we also sequenced 3 pools of samples, one from each sample type, for long reads (LRs) sequencing using the Nanopore PromethION flowcell. Animals were sampled at different life stages to better characterise the diversity of the cattle microbiome (Fig. 1A). This included sampling animals at marking, weaning, before feedlot, during feedlot and at the slaughterhouse. In total, samples from three different cohorts of Australian Angus cattle, originating from two different farms, were collected to generate this comprehensive dataset. Following a pipeline for the recovery of MAGs (Fig. 1B), we recovered a total of 2388 MAGs from the faecal samples, 531 from saliva and 157 from nasal mucus meeting the MIMAG standard of medium quality (MQ, >50% completeness and <10% contamination) to high quality (HQ, >90% completeness and <5% contamination) draft genomes^24^.

**Fig. 2:**
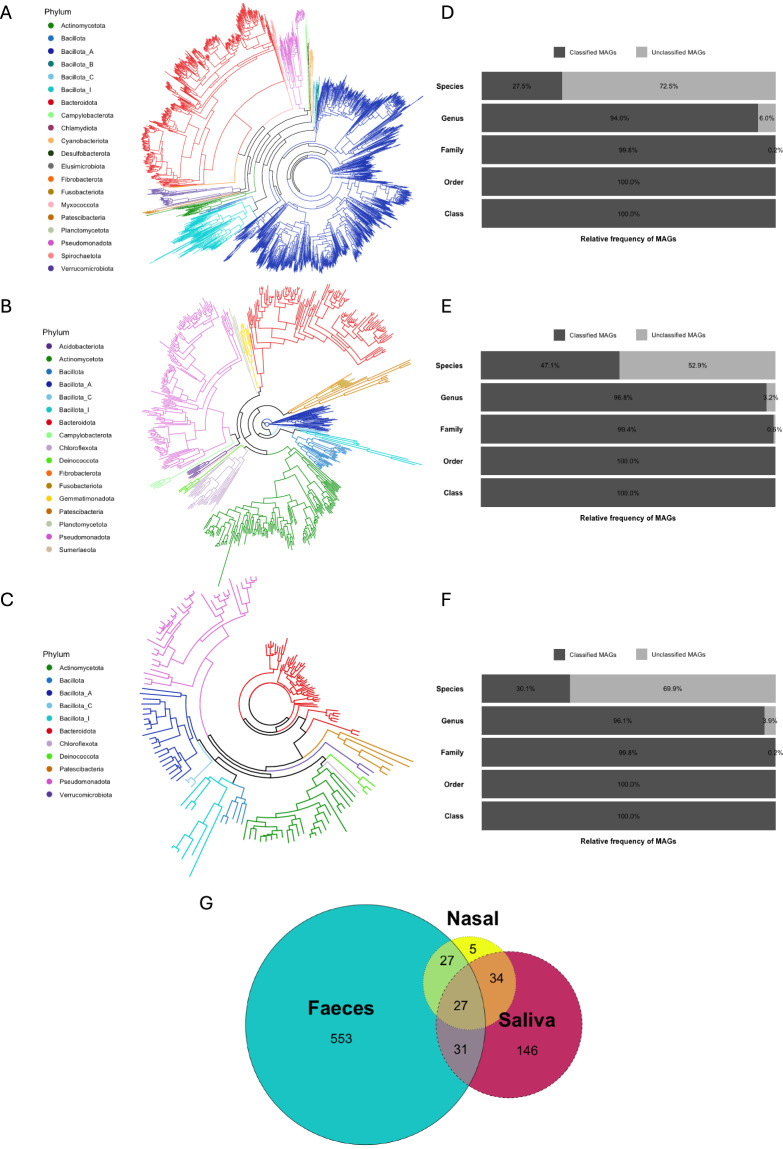
Overview of the MAGs taxonomic diversity. Phylogenetic tree of faecal (**A**), oral (**B**) and nasal (**C**) bacterial species-level MAGs constructed using a concatenated set of 120 conserved bacterial marker genes. Each node was coloured with the bacterial phylum belonging to the associated MAG. Bar graphs representing the frequency of MAGs at each taxonomic level using the GTDB-TK classification from the faecal (**D**), saliva (**E**) and nasal (**F**) samples. Venn diagram depicting the number of shared Genera across all sample types (**G**).

**Fig. 3:**
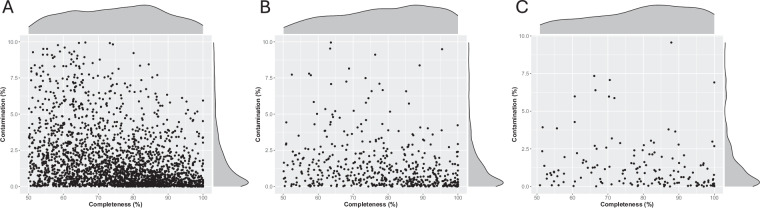
MAGs quality assessment. Genome statistics representing the quality and contamination of MAGs associated with faeces (**A**), saliva (**B**) and nasal mucus (**C**) samples.

Among all MAGs recovered in this study, only 11 from faeces, 3 from saliva and 2 from nasal mucus were classified as Archaea, while the remaining MAGs were assigned to Bacteria. In the faecal samples, Bacillota was the most represented phylum (1662 MAGs) followed by Bacteroidota (516 MAGs) and Pseudomonadota (77 MAGs) (Fig. 2A). In saliva samples, 144 MAGs belonged to Pseudomonadota, 127 to Actinobacteria, 102 to Bacteroidota and 87 to Bacillota (Fig. 2B). Similar phylum representation was also found in the nasal mucus samples where we recovered 50 MAGs classified as Bacteroidota, 37 MAGs as Bacillota, 33 as Pseudomonadota and 24 as Actinobacteria (Fig. 2C). Shared taxonomic levels were observed between MAGs from different body sites. For instance, 27 Genera were shared across all sample types while 553, 146 and 5 were unique to samples from faeces, saliva and nasal mucus, respectively (Fig. 2G). All MAGs were classified at both Class and Order level (Fig. 2D–F). However, some MAGs couldn’t be classified at the Family, Genus and Species levels, suggesting that they are novel bacterial members that have not been characterised yet. In fact, 72.5% faeces MAGs, 52.9% saliva MAGs and 69.9% nasal MAGs couldn’t be assigned to a Species (Fig. 2D–F). Similarities between sample types could also be exemplified by dereplicating all 3076 MAGs at 95% average nucleotide identity (ANI), resulting in 2881 dereplicated MAGs. MAGs associated with the faecal samples had an average completeness of 75% and contamination of 1.7% (Fig. 3A). On average, higher-quality MAGs were recovered from the saliva and nasal samples with 79% completeness and 1.4% contamination, and 80% completeness and 1.3% contamination respectively (Fig. 3B,C).

**Fig. 4:**
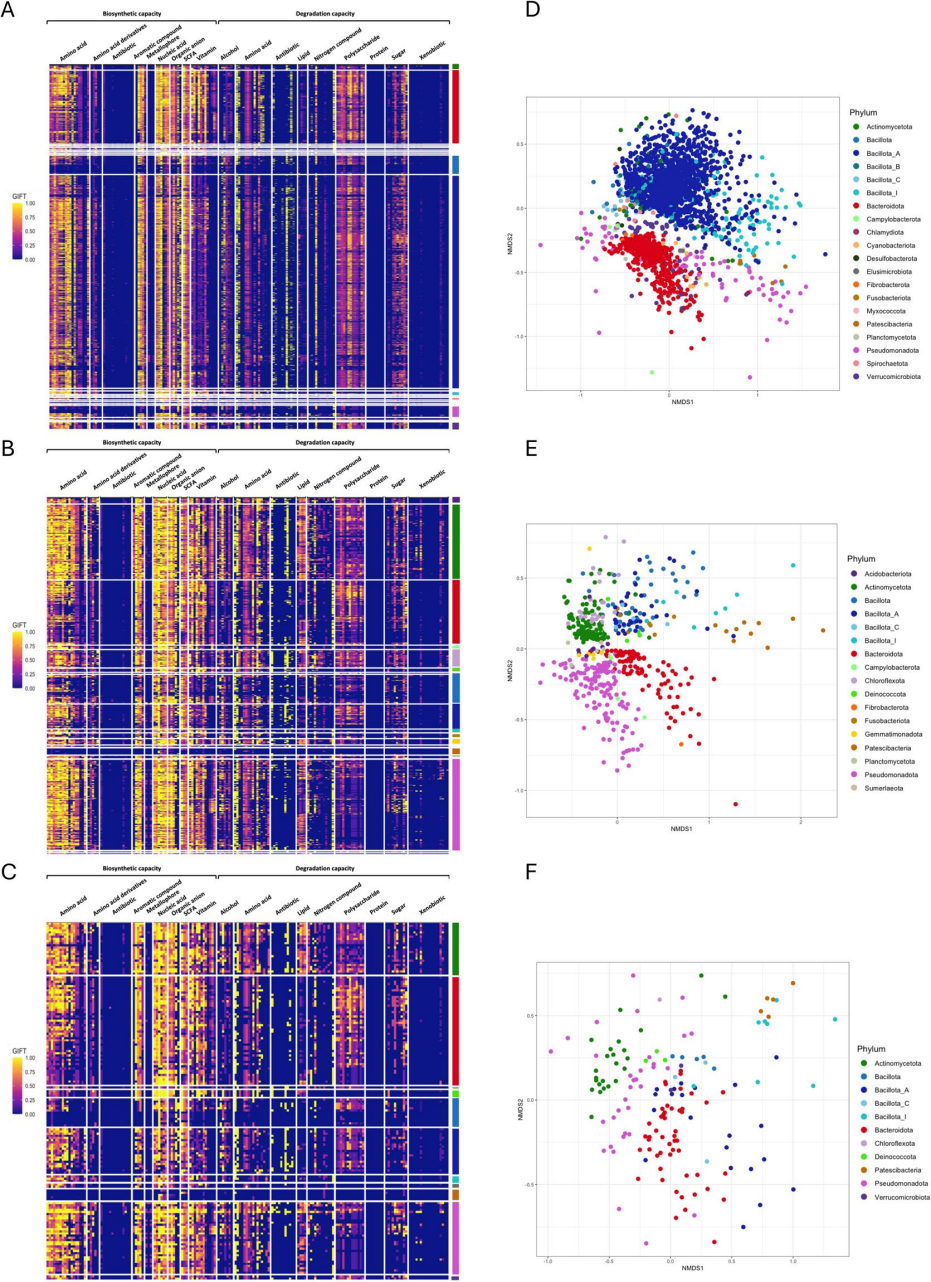
Functional profiling of the MAGs. Heatmaps representing the faecal (**A**), oral (**B**) and nasal (**C**) MAG GIFTs. A 0 value corresponds to no gene present in a functional trait and 1 corresponds to all genes involved in a functional trait being present in the MAG. The ordination plots represent the functional profile of all faecal (**D**), oral (**E**) and nasal (**F**) MAGs at gene level (all genes functionally annotated using DRAM). Colour was assigned based on the bacterial phylum of the MAGs.

The functional potential of MAGs was determined by transforming raw functional annotations from the KEGG^25^ and dbCAN2^26^ databases into basal quantitative genome-inferred functional traits (GIFTs)^27^. This allowed us to explore the biosynthetic and degradation capacity of all generated MAGs. In all sample types, the most abundant pathways were associated with amino acid production, aromatic compound production, nucleic acid production, organic anion production and short chain fatty acid (SCFA) production (Fig. 4A–C). In regards to degradation capacity, pathways related to polysaccharide degradation and sugar degradation were the most abundant, particularly in MAGs assigned to Bacteroidota, Bacillota and Actinomycetota. The functional potential similarities of MAGs from similar lineages are best exemplified in the ordination plots showing stark clustering of MAGs at the phylum level (Fig. 4D–F).

Overall, this study has uncovered substantial novel microbial genomes from multiple body sites of beef cattle. To the best of our knowledge, this is the first report of MAGs recovered from the oral and nasal cavity and faecal microbiome of Angus cattle. We expect our genome database to be widely used as a reference for future studies exploring the functional role of these microbial communities and their interactions with the host. Indeed, this comprehensive dataset will help our understanding of the cattle microbiome in many ways such as in a health and disease context or in relation to animal productivity. Ultimately, these findings will contribute to improving current farming practices.

## Methods

All procedures involving animals complied with the Australian Code for the Care and Use of Animals for Scientific Purposes 2013 8th edition, and ethical approval was granted by the CSIRO Livestock Animal Ethics Committee (ARA 22/13 and ARA 22/32).

### Sample collection

All samples were collected from Australian Angus cattle in 2022 and 2023. In total, we sampled three different cohorts at different life stages which included marking (calves mustered for first vaccination and male castration), weaning (calves separated from the cows), before entering the feedlot (at pasture), during the feedlot (71 days on feed) and at slaughter (Fig. 1A). Faeces were sampled by faecal grab using nitrile gloves and transferred into specimen containers. Containers were then placed on ice for no more than four hours, and later stored at −80 °C until DNA extraction.

Nasal mucus was sampled with threaded sponges normally used for synchronising oestrous in sheep and goats (SYNCRITE-45 vaginal sponge, Animal Health Supplies, Australia). One sponge was placed in each nostril between 5 and 10 cm into the nasal cavity and each nostril was slowly massaged for one minute to let nasal mucus impregnate the sponges. The sponges were then pulled by the thread and placed into 50 mL falcon tube and put on ice. Within 4 hours, paired sponges were transferred into a 50 mL Luer Lock syringe (NIPRO Medical, USA) with sterile tweezers and ~20 mL of sterile phosphate-buffer saline (PBS) was added on top of the sponges. The mix of PBS and nasal mucus was then squeezed out back into the 50 mL falcon tube. A second wash was performed to recover a total of 25–30 mL of solution. Following this, the falcon tubes were centrifuged at 4 °C at 3,275 g for 20 min to generate a cell pellet. The supernatant was then discarded, and the cell pellet resuspended in 3 mL of PBS. This solution was subsequently aliquoted equally into two 2 mL tubes and stored at −80 °C until DNA/protein extraction. To collect nasal mucus from the animals at marking (only one collection from herd 2, Fig. 1A), floq swabs (Copan ESwab regular floq, USA) were used instead of the sponges as their nostrils were significantly smaller than the older animals. We carefully swabbed one nostril up to 15 cm deep into the nasal cavity and placed the swab back into a tube containing 1 mL of Liquid Amies. The tubes were then placed on ice until the end of the sampling and stored at −80 °C for downstream DNA extraction.

To collect saliva, two 10 × 10 cm gauzes (Livingstone, Australia) were attached to the end of a Foerster sponge holding forceps and the entire buccal cavity of the animal was swabbed vigorously for 20 seconds (this includes the top and underneath the tongue as well as the inside of the cheeks). The soaked gauzes were then placed into 50 mL falcon tubes and placed on ice until the end of sampling. Next, around 15 mL of sterile PBS was added to the tubes which was shaken vigorously until the gauzes were fully soaked. The gauzes were subsequently transferred into a 50 mL Luer Lock syringe with sterile tweezers and the liquid containing the saliva and PBS was squeezed out back into the 50 mL falcon tube. A second wash was performed to recover around 25–30 mL of solution. The same protocol as for the nasal mucus samples was followed to generate a cell pellet which was aliquoted into two 2 mL tubes for DNA and protein extractions. Immediately after this, propidium monoazide (PMA, Biotium, USA) was applied to the samples prepared for DNA extraction to reduce host DNA for downstream sequencing^28^. For this, the 2 mL tubes were centrifuged at 10,000 g for 10 min. The supernatant was discarded, and the pellet was resuspended in 200 µL of sterile water by pipetting and a brief vortex. The samples were then left at room temperature for 5 min to osmotically lyse the host cells. 10 µL of 0.2 mM PMA solution was added to the tubes to obtain a final concentration of 10 µM PMA. The tubes were briefly vortexed and left in the dark at room temperature for 5 min. Next, samples were laid horizontally on ice for 25 min at a distance of <30 cm of two LED lights of 4800 lumens. Samples were briefly vortexed every ~5 min. After light exposure, samples were placed at −80 °C until DNA extraction.

### DNA extraction

We used the CTAB method to extract DNA of all samples^29^. However, the DNA input varied between sample types and collection methods. For faecal samples, 200 mg of thawed faeces was used as input for DNA extraction. An extra purification step with polyethylene glycol (PEG4000) at the beginning of the DNA extraction was used for all faecal samples. For nasal swab samples, the tip of the swab was cut at the base to only leave the impregnated swab in a new 2 mL tube and it was used as input for DNA extraction. Regarding nasal samples, the ~1.5 mL aliquot was transferred into a new screw cap 2 mL tube and centrifuged for 15 min at 10,000 g. The supernatant was then discarded, and the cell pellet was used as input for DNA extraction. For nasal swabs, the swabs were transferred into a new 2 mL screw cap tube and the tip was cut at the base of the swab. The impregnated cotton swab were then used for DNA extraction. Finally, for saliva samples, the 210 µL solution was transferred into a new 2 mL screw cap tube and used for DNA extraction.

Briefly, the starting material was mixed with 200 mg of sterile silica-zirconium beads (1:1:1 mixture of 0.1, 0.5 and 1 mm beads) and 800 µL of CTAB isolation buffer. For faecal samples, 120 µL of PEG4000 was added during this step as mentioned previously. Lysis was performed by bead beating at 6.5 m/s for 2 min using the FastPrep24 instrument (MP Biomedicals, USA). Samples were incubated at 70 °C for 20 min and centrifuged at 10,000 g for 10 min. The supernatant was mixed with 0.1 mg of RNAse-A (Qiagen, USA) and incubated at 60 °C for 20 min. An equi-volume of Chloroform:Isoamyl (24:1, Sigma-Aldrich, Germany) was added to the tube, shaken vigorously, and centrifuged at 10,000 g for 10 min. The upper phase was transferred into a new tube with an equi-volume of Phenol:Chloroform:Isoamyl alcohol (25:24:1, Sigma-Aldrich, Germany). The tubes were shaken vigorously and centrifuged at 10,000 g for 10 min. The supernatant was transferred to a new 1.5 mL tube and the DNA was washed with 0.8 volume of isopropanol. The pellet was then washed with 750 µL of 70% ethanol. The DNA was finally eluted in EB buffer (Qiagen, USA) and the yield and purity were assessed using a NanoDrop 8000 spectrophotometer (Thermo Fisher Scientific, USA).

### Library preparation and sequencing

For short reads sequencing, libraries from all samples but 80 were prepared using the Nextera DNA flex kit (Illumina, USA) and sequenced on an Illumina NovaSeq 6000 platform (2x151bp) at the Australian Centre for Ecogenomics (University of Queensland). Libraries from the other 80 samples (all samples from cohort 3) were prepared using the Illumina DNA Prep M kit (Illumina, USA) and sequenced on an Illumina X platform (2x151bp) at the Australian Genome Research Facility (Melbourne, Australia). Pooled samples from the same sample type were used for long read sequencing. Libraries of the three samples (faeces, saliva and nasal mucus) were prepared using LSK-10 ligation preparation method and sequenced using an adaptive sequencing protocol on an Oxford Nanopore PromethION at the Australian Centre for Ecogenomics (University of Queensland).

### Quality control, host removal and assembly

In total, we generated over 3TB of Illumina SR sequences. This included 1.3TB of sequences from faeces, 996GB from saliva and 988GB from nasal mucus samples. For Nanopore LR sequencing, the fast5 data was converted to fastq format using Guppy (v.7.0.9). This yielded a total of 229GB sequences including 99GB from faeces, 81 from saliva and 48 from nasal mucus samples.

The quality of Illumina sequences was initially checked using MultiQC^30^ (v1.19) and the sequences were processed using Trim Galore v0.6.10 using the following parameters:–paired –fastqc–length 40. SR reads were mapped to the Bos Taurus genome using bbwrap from the BBTools suite v39.06 with standard parameters (https://ftp.ensembl.org/pub/release-108/fasta/bos_taurus/dna/Bos_taurus.ARS-UCD1.2.dna.toplevel.fa.gz). LR reads were mapped to the host genome using minimap2^31^ (v.2.25) using -ax map-ont parameters. The resultant sam files were then converted to fastq files using samtools^32^ with -n and -f 4 parameters. The unmapped reads were concatenated into one file by sample type, collection point and sequencing type. Considering the huge amount of data for some of the fastq files, reads were normalised to remove excess coverage before running the assembly using bbnorm with the following parameters: target = 70, min = 2, prefilter = t. This step was performed for 4 collections: faeces collected before feedlot from cohort 1, saliva collected before feedlot from cohort 1, faeces collected at marking from cohort 2 and saliva collected at marking from cohort 2. These files were then used to generate assemblies. In total, we generated 25 assemblies (22 from SR and 3 from LR). SR assemblies were constructed using metaSPAdes v3.15.5 with default parameters^33^. LR assemblies were generated using metaFlye^34^ (v2.9.3) with the following parameters:--nano-hq –meta. LR assemblies were then polished using one round with Racon v1.4.21^35^ and two rounds with medaka v1.4.4 using default parameters. LR and SR from specific nasal samples were also used together to generate an assembly with metaSPAdes using default parameters. Assembly using both SR and LR were not performed for the saliva and faeces samples due to computational resources limitations.

### Genome binning

First, contigs coverage from all assemblies was calculated using Bowtie2^36^ (v.2.4.4). This consisted of building an index using the –large-index flag, mapping the reads back to the assembly using the -q –sensitive-local parameters, converting the sam file into bam file and finally sorting and indexing the bam file using default parameters. Both, MetaBAT2, MetaDecoder and SemiBin2 were used to generate bins^37–39^. For MetaBAT2, the script jgi_summarise_BAM_contig_depth was used to create a depth file prior running the main command with the -m 1500 parameters to only use contigs >1500 bp. For MetaDecoder, coverage was first calculated using the MetaDecoder coverage command and using the sam file as input. The clustering command was then ran using the –min_sequence_length 2000. For SemiBin2, the sorted bam file was used to generate bins using default parameters. Bins obtained from the three different tools were then refined using MetaWRAP^40^ (v.1.2) with the bin_refinement module with the following settings: -c 50 and -x 10. This step further improved the quality of the bins by using the best bins from the three different tools and keeping only bins with a completeness >50% and contamination <10% using CheckM^41^ (v.1.0.12). All filtered bins generated from the different assemblies were then pooled by sample type. To assess the quality of the filtered bins, CheckM2^42^ (v.1.0.2) was performed using the checkm2 predict function. As CheckM2 uses a different algorithm than CheckM, some remaining bins didn’t pass the >50% completeness and <10% contamination threshold and were therefore removed from the dataset. Bins were dereplicated at 95% ANI using dRep^43^ (v.3.4.0) to ensure that only unique species level genomes remained in the final dataset.

### Taxonomic assignment and functional annotation

To assess the taxonomic classifications of the MAGs, the function gtdbtk classify_wf from GTDB-Tk (v.2.1.1) was used with the–full-tree flag and the reference database GTDB release 220. The function gtdbtk de_novo_wf was used with the flag–outgroup_taxon p__Acetothermia. The generated multiple sequence alignment (MSA) file with the user genomes was then used to create a phylogenetic tree using FastTree^44^ (v.2.1.11) which was visualised using TreeViewer^45^ (v.2.2.0). Gene prediction and annotation was performed using DRAM^46^ (Distilled and Refined Annotation of Metabolism) v1.5.0 using default parameters. GIFTs for each MAGs were generated using the distillR package (https://github.com/anttonalberdi/distillR) using the function distill.

## Data Records

The raw shotgun metagenomics reads and the sequence data for the 3076 MAGs have been deposited in the National Center for Biotechnology Information (NCBI) database under the Bioprojects PRJNA1225320 and PRJNA1227551 as Sequence Reads Archive (SRA)^47,48^. In addition, the sequence data of all MAGs have been deposited to the Figshare database^49^.

## Technical Validation

DNA extractions were performed by batch from the same sample type to avoid cross contamination between different sample types. All DNA samples were assessed for quantity and quality using a NanoDrop 2000 Spectrophotometer and the DNA was again checked using a 4200 TapeStation system (Agilent, USA) prior to the generation of libraries. Blank controls were sequenced to check for potential contaminants during the laboratory work. Sequences were quality checked using Trim Galore to remove contaminants bases, adapter sequences and short length reads. In adherence with the MIMAG guidelines, all MAGs recovered in this study were assessed for quality and contamination using both CheckM and Checkm2 and only MAGs meeting the specified thresholds were kept in the final dataset.

## Data Availability

All open-source software used in this study including the versions and parameters (when not using default settings) are described in the manuscript and referenced in the methods section. No custom scripts were used during this study.
